# Reducing risks in complex care transitions in rural areas: a grounded theory

**DOI:** 10.1080/17482631.2023.2185964

**Published:** 2023-03-03

**Authors:** Idun Winqvist, Ulla Näppä, Helén Rönning, Marie Häggström

**Affiliations:** Department of Health Sciences, Mid Sweden University, Östersund and Sundsvall, Sweden

**Keywords:** Care transitions, discharge, grounded theory, home healthcare, hospital care, inter-organizational cooperation, nursing, rural health services

## Abstract

**Purpose:**

Although previous research indicates that care transitions differ between rural and urban areas, the knowledge of challenges related to care transitions in rural areas appears limited. This study aimed to provide a deeper understanding of what registered nurses’ perceive as the main concerns in care transitions from hospital care to home healthcare in rural areas, and how they handle these during the care transition process.

**Methods:**

A Constructivist Grounded Theory method based on individual interviews with 21 registered nurses.

**Results:**

The main concern in the transition process was “Care coordination in a complex context”. The complexity stemmed from several environmental and organizational factors, creating a messy and fragmented context for registered nurses to navigate. The core category “Actively communicating to reduce patient safety risks” was explained by the three categories– “Collaborating on expected care needs”, “Anticipating obstacles” and “Timing the departure”.

**Conclusions:**

The study shows a very complex and stressed process that includes several organizations and actors. Reducing risks during the transition process can be facilitated by clear guidelines, tools for communication across organizations and sufficient staffing.

## Introduction

Nursing means safeguarding care quality and patient safety (International Council of Nurses, 2021) and registered nurses (RNs) are well-positioned when it comes to ensuring the continuity of care in transitions between different healthcare organizations (Östman et al., 2021). Care transition (CT) refers to the patient’s movement from one care provider to another, such as between hospital and home healthcare (World Health Organization [WHO], 2016). It is rather a process than a specific point in time (Geary & Schumacher, 2012). Hospital stays are often short (Petersen et al., 2019) and healthcare providers value rapid discharges (Neiterman et al., 2015). CTs from hospital to home have been identified as risky processes concerning patient safety and the elderly with complex conditions are most at risk during transitions (Campbell Britton et al., 2020; Markiewicz et al., 2020). RNs have a key role in CTs and interact with patients and relatives in their most vulnerable situations (Camicia & Lutz, 2016), such as during CTs (WHO, 2016).

People aged 65 or more represent the majority of patients readmitted within 30 days after hospital discharge (Åhsberg, 2019) and this specific population is estimated to increase by a third in the next thirty years (King et al., 2021). Older people often have convoluted care needs not only due to their medical conditions but also their cognitive state, which increases the demands on environmental assets (Greenstein et al., 2019). An environment like the patient’s home strongly influences CTs (Carman et al., 2021; Zwart et al., 2021). The risk of readmissions is higher when discharged to home healthcare in an ordinary residence than to a skilled nursing facility (Werner et al., 2019). Successful discharges and the risk of readmissions within 30 days of discharge differ between urban and rural residents (Anderson et al., 2021).

Frail older people tend to experience transitions from hospital to home as more unsafe and troublesome than less frail people (Andreasen et al., 2015). The organization of CTs from hospital to home is a confusing topic for the elderly to familiarize themselves with, placing them in vulnerable positions (Hvalvik & Dale, 2015; Neiterman et al., 2015). In a study with no focus on the urban/rural- perspective, patients experienced emotional and practical challenges shortly after the CT from the hospital (Neiterman et al., 2015). The quality of CTs affect not only the patients being transitioned but also their relatives (Häggström et al., 2014; Pulst et al., 2019) who wish to be active participants when care is transitioned from hospital to home (Hvalvik & Reierson, 2015). According to Lindblom et al. (2020), patients and their relatives may experience discharge from hospital as something stressful that they are forced into without warning, which further emphasizes the importance of the quality of CTs to them.

Despite the knowledge that CTs are risky and affected by the environment, few previous studies (Magilvy & Congdon, 2000; Magilvy et al., 1994) have been found presenting RNs’ perspectives on the CT from hospital to home in ordinary residences, focusing on rurality in either analysis or discussion. Increased knowledge of RNs’ perspective on the CT process could contribute to further improvements in the practice thereof and policies for the same, for the benefit of patients and their relatives. Thus, this study aimed to provide a deeper understanding of what registered nurses’ (RNs’) perceive as the main concerns in care transitions (CTs) from hospital care to home healthcare in rural areas, and how they handle these during the care transition process.

## Method

### Study design and philosophical assumptions

A Constructivist Grounded Theory approach, as described by Charmaz (2014) was used, as it is closely linked to the ontological and epistemological assumptions of the authors. The view of knowledge and reality can differ between different variants of Grounded Theory. Humanistic researchers’ world perspective is often revealed through their humanistic methods, and interpretive researchers who use qualitative methods see reality as a human construction. Reality is subjectively experienced, relative, and socially constructed whereby researchers interpret data based on their own social realities (Plummer, 2011; Tuli, 2011). Theory is co-constructed in an interplay between the participants’ statements and the researcher’s interpretations. It is based on collected data but recognizes the researcher’s subjective role through the collection and interpretation of data, which in turn are formed by past and present parts of the world under study (Charmaz, 2014). Knowledge is relativistic, created between the observer and the observed with an interpreted understanding of the subject’s meanings. Truth is thus dependent on one’s perspective rather than single, realistic and lasting, while reality is regarded as *one* reality rather than *the* reality (Lomborg & Kirkevold, 2003). This study is based on interviews with RNs. Being an interviewer means being the main instrument in this study, which entails a need to critically examine one’s pre-understanding and its potential impact in order not to influence research in a negative sense, by being reflexive (Patton, 2015). While handling the data, the authors’ preconceptions were sought to be bridled through critical discussions, to avoid negatively affecting the result through the impact of previous experiences or by misinterpreting the data. In addition to joint discussions regarding own pre-understanding, the first author wrote an analytical and methodological research journal which was shared with the other authors, as Charmaz (2014) recommends for maintaining reflexivity.

### Setting, participants and procedure

This study was conducted in Sweden, where CTs from hospital to home healthcare is regulated by the *Act on cooperation in the event of discharge from inpatient health care* (SFS, 2017a, p. 612). Whether the responsibility for outpatient healthcare belongs to the municipality or the county council’s primary care centre is regulated in a regional collaboration agreement between the county council and the belonging municipalities, which also regulates that inter-organizational communication concerning CTs should occur in a digital e-message system. In this study, home healthcare refers to healthcare provided in ordinary housing, i.e., the patient’s private residence, which is where most municipal care is provided (SOU, 2020, p. 80). Municipal care in ordinary housing constitutes two types of care: home healthcare based on the Health and Medical Services Act (SFS, 2017b, p. 30) and home care services based on the Social Services Act (SFS, 2001, p. 453).

As there is no ideal definition of rural (Hawley et al., 2016), the authors wish to describe the context of the conduct of the study. The Swedish Agency for Growth Policy Analysis (2014) defines the municipalities where this study was carried out as where the majority population has at least 45 minutes and an average of 90 minutes travel time to an area with a population of at least 50,000 inhabitants. Participating RNs stated that the patients could live 80–250 kilometres from the county hospital responsible for their inpatient care. The RNs in home healthcare could have a distance of at most 20–150 kilometres between their office and the patient’s home.

Similar to many other Grounded Theory studies, initial sampling in this study was purposive (Chun Tie et al., 2019)–participants were recruited if they were considered able to contribute to the understanding of the studied CT process. A total of 21 RNs participated; their demographics are illustrated in Table 1. All had a bachelor’s degree in nursing, while some had also completed master’s education for specialist training in district nursing, elderly care, oncology, or medicine. Criteria for inclusion were that the RNs were employed either in a somatic care department at the region’s county hospital or in home health care in one of six rural municipalities. Another criterion for inclusion was that they had experience of care transfers from hospital to home health care in sparsely populated areas.

**Table I.** Overview of participants’ demographics.

Participants	Age	Sex		Specialist trained RN	Years of service as an RN
		Female	Male		
RN at the hospital (*n*=8)	26–50 y (md 38 y)	7	1	1	2–25 y (md 9,75 y)
RN in home healthcare (*n*=13)	31–64 y (md 51 y)	13	0	13	6–30 y (md 18 y)

Approval for the study was obtained from the hospital clinic managers and the home healthcare managers, whereby they forwarded written and videotaped verbal information about the study to the RNs as an offer to participate. One hospital clinic manager declined participation due to the high workload in the wards. The forwarded information included the voluntariness of participation, the freedom to withdraw their participation at any time without explanation, and a promise of pseudonymisation. All participants signed an informed written consent form before participating in the study.

### Data collection

Before data collection, all authors jointly designed an interview guide containing open-ended questions (Charmaz, 2014; Patton, 2015). This was evaluated through two test interviews with RNs (Kallio et al., 2016). Since these RNs had not worked at the county hospital for several years, their memories were vague and related to prior guidelines for inter-organizational collaboration. One of these RNs had previously worked in home healthcare but not in a rural municipality. These test interviews were therefore not included in the results but enabled clarification of one question in the interview guide before the interviews were conducted.

The individual interviews were conducted in August-October 2021 by the first author, who presented herself as a PhD student with work experience as RN. The interviews took place by participants’ choice either face-to-face (*n* = 10), by telephone (*n* = 2), or through Zoom or Teams (*n* = 9). They lasted between 63 and 149 minutes (md = 83 min). To openly explore the participants’ personal experiences in the research topic, the first question asked was “How does CT from hospital care to home care take place?”, followed by questions such as “Can you please tell me about an occasion when a CT went less than well?” and “How do you work as a nurse to make the CT as good as possible in a rural environment?”.

Following the Grounded Theory method, theoretical sampling was used to seek additional data to expand and refine the theory evolving during the analytical process, by asking additional interview questions, widening the scope of existing questions and recruiting participants with additional diversity of attributes within the same group (Ligita et al., 2019). The theoretical sampling was guided by the aim of theoretical saturation, the stage where the same instances appear again and again and no new properties of the categories are found (Charmaz, 2014; Saunders et al., 2018). It focused partly on equalizing the views of RHHCs and RHs, partly on adding questions to deepen the understanding by comparing variations in the experiences. After interviewing 16 people and adding questions about emerging events and actions, the properties of the categories were perceived as saturated, yet another 5 participants were interviewed to ensure that this was the case. Examples of added questions are: “Who is responsible for the patient during transport from hospital to home?” and “What do you do as a nurse when you discover that a transition has not gone well?”. To further understand the CT process from the participants’ perspectives and to saturate the categories, data collection was thus affected by the parallel ongoing analysis, as the main concern, the core category and the categories emerged.

Probing questions were asked to encourage participants to explain their views on events and to learn and gain further understanding of their perceptions and experiences. A probing question could be “If you didn’t act that way, what do you think it could lead to?”. Interviews were audio-recorded using a mobile phone, transcribed verbatim by the first author and read by the other authors. During the transcription which took place shortly after the interviews, the participating RNs were pseudonymised with a number (P1-P21) and the workplace (H=hospital, HHC=home health care). The transcriptions were compared with the audio recordings to ensure their validity. Besides audio recording, notes were taken during the interviews to facilitate returning to previously discussed aspects and asking more about them (Charmaz, 2014). All participants declined the offer to share with them the material for correction if needed, which within constructivist epistemology can be a procedure for member checking of transcripts (Birt et al., 2016; Morse, 2015).

### Data analysis

**Figure 1.**
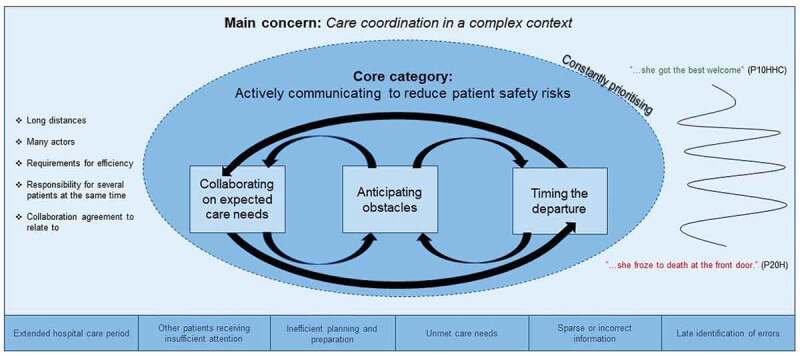
An illustration of care transitions from hospital care to home healthcare in rural areas, constructed from a nursing perspective. The substantive theory “Reducing risks in complex care transitions” show how RNs tried to achieve their main concern Care coordination in a complex process, doing so by the core category of Actively communicating to reduce patient safety risks. The three interacting categories explains actions of particular importance in order to reduce risks.

The analysis was driven by the search for the core category, of which building blocks could be glimpsed during the preliminary coding of early interviews. The initial coding of actions was done by the first author, where lines and segments received shorter labels kept close to the original text. Memos were written during all stages of the research, which contributed to developing ideas both in analysis and in preparation for subsequent interviews (Charmaz, 2014). The question “what is going on here?” was asked throughout to understand the participant’s actions and meanings. The most salient initial codes led to a more focused coding. The constant comparisons of the focused codes led to sections of data, which, during a theoretical coding phase, were labelled and drawn in diagrams and figures to better understand their *inter se* relationships and connections. The diagramming and drawing were helped by the memos, making comparisons and enabling the theoretical coding surrounding the core category and categories, visualized by the circle in Figure 1. The data were synthesized and organized and the focused codes returned to a whole, driven by the questions “how”, “who” and “why”, to understand the participants’ perspectives and social processes (Charmaz, 2014). The core category, along with the main concern and categories, was constructed throughout the whole process of interviews and analysis. These were formulated and reformulated as the theory became clearer.

The analysis involved a movement between empiricism and theory and meant going back and forth between the data. The research process could be interpreted as linear as described in this section, but in fact, involved a constant comparative movement between codes, categories, and text in the construction of the core category. This movement was supported by the analytical memos. Together with the data, codes and categories, they formed the basis for the analytical discussions.

The discussions between the authors led to questions about the categories building the core category, whose answers were found through further investigations of the data, contributing to several movements in the iterative analytical process. The categories were considered saturated when the authors had no more questions to ask of them and no new properties of theirs were found. This was at the final stage of the analysis, which also included carrying out a systematic literature search to compare the categories against previous theories in the field (Charmaz & Thornberg, 2021). As recommended by Morse (2015), no member checking of the analysis was carried out. The quotes have been carefully translated from Swedish to English, to conform as best as possible to the participants’ versions.

### Ethical considerations

The study was conducted following the Helsinki Declaration (World Medical Association, 2013) and the Ethical Guidelines for Nursing Research in Nordic Countries (Northern Nurses’ Federation, 2003). All data were handled following GDPR (Regulation EU, 2016). According to Swedish legislation (SFS, 2003, p. 460), this study does not require an ethical approval, but and an advisory opinion for the study was still obtained from the Ethical Review Agency in Sweden (Dnr 2021–02578) to ensure that sample recruitment, data collection and handling of data did not contravene good research practice. The participants’ integrity and self-determination were highly valued during the conduct of the study and was a reason why they themselves were offered to decide the time and place for the interview.

## Results

The main concern in transitions from hospital care to home healthcare in rural areas was *Care coordination in a complex context*. The complexity involved several environmental factors concerning both organizational and rural-specific matters. These contributed to a messy and fragmented context for RNs to navigate. The process required many actors, placing demands on personnel resources, which was considered both complicated and necessary. The long distances between the care providers were perceived to create additional pressure in the CTs. These distances meant a prolonged time before any mistakes could be corrected. CTs were rarely perceived as flawless and there was nervousness and anxiety among RNs about the outcome of the transition, which stemmed from past experiences and out of goodwill for the patient’s well-being.

The feeling is that with the heart in the pit of the throat that

*gasps for air* it went well(P4H)

There was also an anxiety among the nurses stemming from organizational factors. The collaboration agreement created uncertainty because it could be interpreted in different ways. There was a consensus among the RNs that the care processes were fast and needed to be so, as long as patient safety was not negatively affected whereby also time for planning was required. The pursuit of patient safety was one reason for the pursuit of rapid care transitions. The hospital environment was not considered optimal to stay in for a fully treated patient who would be better off at home, as it entailed, for example, a higher risk of infections than a home environment. One prominent organizational aspect concerned the lack of beds at the hospital, necessitating the keeping of inpatient care periods as short as possible because new patients in need of emergency medical care needed the bed. This created stress in balancing the needs of the patient being transitioned, other patients in the ward and acutely ill patients who would next need a bed. Further, the workload at the hospital meant that RNs prioritized emergency medical care before discharge planning for stable patients.

*The core category Actively communicating to reduce patient safety risks* was the way for RNs to handle the main concern. The communication took place between all actors involved in the patient’s care at the hospital and those expected to be involved in the patient’s care after discharge. Foresight was important when reducing risks in rural areas due to the distance’s impact on time and for allowing time to plan and prepare. It reduced the risk of patient suffering and prevented having a more strenuous work situation later.

So that we get to know in good time, so that there are not only emergency solutions, that, that is like the best with creating patient safety. (P2HHC)

Actively communicating meant being quick, clear and one step ahead. The core category was considered central to quality-of-CTs. When the CTs went well, it was attributed to well-functioning communication, the RN in home healthcare (RNHHC) could assume responsibility for care having no questions unanswered. Correspondingly, on the occasions when care transfers were not successful it was often considered to be devolve upon poor communication.

So it’s about acting quickly and asking questions that are adequate for my task. And then I think the transition will be better in all aspects. It is communication, the world’s most difficult mission, that is at stake in ALL situations. (P10HHC)

Actively communicating included actively observing conversations between other actors in the digital e-message system. Without clear communication, RNHHCs would not feel safe being responsible for the patient after discharge. Checking the digital e-message system more often than agreed was a way for RNHHCs to reduce the risk of being unable to meet the care needs in the event of quick decisions about discharge. However, checking it more often than agreed was difficult for the RNHHCs. Due to long distances, they spent many hours driving to get to their patients. Delayed response to messages in the digital e-message system was a source of frustration since the transition process was supposed to be rapid. Even though RNHs wanted quick responses to messages, they considered the time spent in the digital e-message system after actualizing the patient to be an incorrect priority. They thought they should reduce risks by focusing on acutely ill patients in the ward rather than the stable ones who awaited completed discharge planning.

Even though the RNs were referred to communicate through the digital e-message system, written communication could not replace the need for verbal communication by phone. This need depended on how well the written communication went and how much was new to the RNHHC. In the event of quick decisions about discharge, RNHs called to speed up the process because they did not have time to wait for written answers and wanted to ensure the information reached the RNHHC in time.

If you have someone who really needs to go home, they might be waiting for something, they can’t go home without intermittent catheterization or medication, help with medication or something else, and you get no answer. Then it might be nice to call them. (P16H)

By speeding up the process this way they were able to reduce the risk of additional days as hospitalized and thereby occupying a bed from another patient in need of emergency care. Having talked to each other meant knowing that the information was received, which gave a sense of security. Verbal communication was considered to offer more and different information than written one, such as advice on nursing strategies like how to respond to cognitively impaired patients. It gave RNHs information about the patient’s habitual condition, which was rarely detailed in the digital e-message system. However, the need for contact by phone was considered to entail challenges like some rural areas not having coverage everywhere. RNHs also perceived phone numbers as inaccessible, as there were no guidelines for phone contact. If the patient had home healthcare previous to the inpatient care period, a phone number could sometimes be in their medical record, or they could ask the patient for it. Otherwise, they had to spend time chasing phone numbers.

The RN’s actions to reduce the risks in the process through active communication are further explained by the *categories Collaborating on expected needs, Anticipating obstacles* and *Timing the departure.*

### Collaborating on expected care needs

Collaborating on expected care needs involved several assessments, interactions and associated potential risks. It explains striving to be one step ahead in the rapid inpatient care process while having to make difficult assessments and anticipating the patient’s future care needs, in the short and long terms. Actualization of the patient in the digital e-message system was considered by the majority to be the starting point of the CT. Doing so early was desired since RNs knew that planning and preparation take time, parallel to the inpatient care periods being short. Otherwise, there was a risk of limited time for communication and planning before the patient was medically fit for discharge, or it would cause a need to extend the care period to do this. To reduce this risk, RNHHCs could actualize the patient even before the patient reached the hospital if the patient had home healthcare before the hospital admission. Regarding those who did not, RNs at the hospital (RNHs) began asking for the patient’s consent to discharge planning at the slightest suspicion of the patient needing care at home after discharge. The patient’s own assessment of future care needs after discharge was important, as the patient’s consent was a prerequisite for discharge planning. The RNHs thus needed strategies for obtaining it. They gradually stepped up from informing about opportunities or asking on several occasions, to be brutally honest with the potential risks seen in discharging the patient without discharge planning. They tried to inform about the benefits of smaller interventions, such as security alarms or food distribution, to get consent to open communication with the municipality and to introduce the patient to the municipal care system. The RNHs thought they, more than the patient, could identify the risks of being discharged without discharge planning. It could seem that the patient did not know his or her own best interests or lacked sufficient insight.

It can be frustrating to discharge a patient where you know that yes, but we’ll see each other again soon because this is not going to go well…(P4H)

Beginning this at an early stage was important since obtaining the patient’s consent could be a time-consuming sequence of the CT process. Going from independence to receiving care at home could, according to the RNHs, be difficult for the patients to accept. Despite desire for rapid transition processes, the patients might need time for it to sink in. Patients who were judged to require help but refused put RNHs in an ethical dilemma, trying to reduce risks of patient suffering and re-enrolment. It felt like a nervous and insecure discharge if they failed to convince the patient, especially if the patient lived in a geographically isolated place.

Above all an ethical dilemma, because everyone wants the best for the patient. Eum, and it doesn’t feel safe and secure to send them home, so it’s not fun, when you see the need exists. (P11H)

It was considered positive when patients were able to actively participate in the discharge planning. The RNHs sought the patient’s wishes about care after discharge but due to the number of patients in the ward, they thought they had no time to ensure the patient’s involvement in the discharge planning as they would have liked. RNHHCs used to inform about the meaning of home healthcare after the patient came home since they did not believe that RNHs had sufficient knowledge about it to be able to inform the patient before discharge.

A preliminary discharge date was mandatory for enrolment in the digital e-message system, whereby it would be appreciated early in the inpatient care process. It constituted a deadline to facilitate the preparation, planning and organization of the CT and it was the physicians’ responsibility to determine. According to guidelines, it would hence be raised daily at rounds, but it was forgotten since it was not on any priority list and the RNHs needed to find routines about it. They could not afford to slow down the process by waiting for the next rounds. Consequently, they had to guess by comparing with previous experiences when they registered the date in the digital e-message system. Both RNHs and RNHHCs regarded it as an unrealistic date because no one could predict what would happen during the period of hospital care.

It can go faster; it can take longer. If the patient suddenly gets a care-related injury, fall event, infection, or something like that, then that preliminary date will not be correct anymore, then you must postpone it. But it is a well-qualified guess. (P9H)

Setting a date soon to postpone it gradually and often, one or two days at a time, was the usual way to handle this difficult task. It was a way of reducing the risk of the patient remaining at the hospital when medically ready for discharge and thus occupying a hospital bed without requiring emergency care. Although the preliminary discharge date was often unrealistic, the RNHHCs used it to calculate. They understood that the final discharge was approaching when RNHs stopped postponing the date.

We always check the planned discharge date when we go into the digital e-message system, to see if they postpone it. Because when they stop postponing it and such, then we understand that she is on her way home. (P10HHC)

The RNHs were to handle the challenge of giving a clear picture of the patient’s activities in daily life (ADL). This formed the basis for welfare officers’ decisions about granting home care services and RNHHCs’ decisions about granting home healthcare. RNHs thought there were no clear guidelines about who in the ward was expected to write the assessment; thus, various professionals did. There were risky aspects to be considered when assessing ADL, perceived as specific to rural areas e.g., the patient’s reduced ability to collect wood outdoors for house heating or shovel snow for the accessibility of the home care service staff. It also involved calculating the risk of not getting help fast enough if the patient was locked out, fell, or was in pain.

It’s just that when there’s a longer distance, it’s a bit lonely, and should you go out, like, or if something happens, it could be that they go outside the door and lock themselves out […] would it be the other way around if you live next to the hospital, then the person who has stopped working will see that this one is on the sidewalk, like it’s more the exposure you get worried about. (P15H)

Not taking these rural-specific aspects into account could risk patients suffering after discharge. However, these risks were by RNHs estimated to be lower than those that could result if the acutely sick patients in the ward did not receive needed care. Thus, they felt compelled to prioritize other tasks that no one else could do, such as caring for more acutely ill patients than those preparing for CT. It was therefore a common solution to let the assistant nurses make the assessments on their behalf.

Other patients need my skills. The specifics go before ADL assessments because no one else can do it. (P4H)

However, RNHs thought it would have been better if they assessed ADL themselves because the assistant nurses’ assessments were not as extensively documented. The foremost assessment the RNHs made was whether the patient could manage his medications, which, according to the RNHHCs, was the physician’s task. When the assessments written by various professionals differed, this confused the RNHHCs as to which one was right.

RNHHCs had to determine through the digital e-message system whether it was reasonable for the patient to be discharged to the conditions of the current home environment. These assessments were facilitated by their access to the hospital’s electronic patient record through the digital e-message system. This access also facilitated their calculations on whether the preliminary discharge date was reasonable. It was based on the patient’s status, probable care needs after discharge and resources available in home healthcare. The remote assessments reduced the risk of an unsafe return home, such as if the patient were to be discharged to an environment that lacked sufficient resources to meet the healthcare needs.

We often have to slow down and say, no, wait, you can’t send them home in this condition to this place! Because it may be two hundred kilometres from the hospital and one hundred kilometres from RN in home healthcare. (P10HHC)

Another remote assessment by RNHHCs was whether the patient qualified for home healthcare. The collaboration agreement regulating the responsibility of the patient’s healthcare between home healthcare, or the primary care centre was perceived as difficult to interpret. RNHHCs perceived that the welfare officer’s decision according to the Social Services Act regulated their decisions on home healthcare, according to the Health and Medical Services Act. One criterion for home healthcare was needing help to eat or drink but the extent of help was not perceived to be clearly stated. The unclear criteria brought an unclear division of responsibilities between the organizations, which led RNHHCs to sometimes deviate from the collaboration agreement to save time spent arguing. Time spent on arguing could otherwise mean for the patient extended hospital stay as medically ready for discharge. They then granted home healthcare, even though they considered the patient did not meet the criteria. This was justified by protecting the patient from being affected by disputes between the two organizations. In this way, they reduced the risk of the patient being discharged without home healthcare despite the need for it due to difficult-to-interpret criteria or insufficiently assessed or documented needs.

The patients should not get in trouble regardless of who is responsible, who will do, who will stand for, who is going here and who is going there. Okay, I guess I’ll have to go and do it so we’ll start somewhere. It will be so. The patient should not suffer. (P17HHC)

### Anticipating obstacles

This category explains, in a tight inpatient care process, striving to be one step ahead, taking time to prepare so that everything is ready and in place when the patient leaves the ward for home. RNHHCs tried to be one step ahead in ensuring the right knowledge and competence in home healthcare to meet the patient’s needs after discharge. Long distances in the countryside entailed the need to delegate more tasks to the assistant nurses in the home care services than if the distances had been shorter. This preparation involved refreshing the knowledge of both RNHHCs and assistant nurses to meet the needs of patients after discharge. Refreshing the knowledge felt safe and positive, although sometimes, the need for it extended the length of the inpatient care period.

If I see that I need to learn this and that, then I can go to the hospital and learn before that discharge date. And I have time to prepare the staff. And I think a lot with the staff, this delegation thing, the knowledge needs to be refreshed. They have done it before but I kind of have to update them on how to do it. (P14HHC)

Insufficient staffing in the home care service otherwise risked extending the inpatient care period, blocking a hospital bed without needing emergency care. Staffing could be challenging in small rural communities since there were only a certain number of inhabitants to employ. Additional challenges were that some assistant nurses did not want to care for the patient because they were related. The small number of inhabitants in the rural communities could also mean advantages such as having prior knowledge of the patient, even if it were not a home healthcare patient before the hospital stay. Frequent healthcare needs, such as intermittent catheterization six times a day or insulin injections four times a day, were sometimes impossible to meet with too few assistant nurses and the long journeys by car to get to the patient’s home. The RNHHCs needed to get an early idea of the patient’s needs because of their dependence on assistant nurses.

*As a nurse, we can’t go and intermittently catheterize someone that often. And it takes a while to delegate staff to that too […] such cases also occur. That you want to bring home people who live as they do quite far away, and they need certain things. And it is almost impossible.* (P19HHC)

At the ward, RNHs way of anticipating obstacles were by ensuring to an even greater extent that everything was in place when the patient was resident in a rural area. This was a way of reducing the risk of any medicine or aid being left when returning home, causing non-compliance in the form of days without medication or increased costs for taxis for delivery or extra orders from pharmacies. It was otherwise considered difficult to solve afterwards because of the long distances. RNs in home healthcare could have the pharmacy 60 kilometres away and up to a week between deliveries of orders. The pharmacies, in turn, had a limited supply, which could lead to even longer delivery times if they had to make an order.

… you may really secure both aids and medicines to a greater extent, because they may have difficulty getting to the pharmacy or pick them up yourself, that you really make sure … (P20H)

However, being early at the ward was difficult. The number of patients per nurse had not decreased in line with increased care needs, and the prioritization of the acutely ill patients rather than the stable ones to be discharged risked things being missed. As a resource, RNHs could use a pharmacist or a colleague to prepare the dosette box that would accompany the patient home. An additional way to prepare was to put the medicines into bags instead of sorting them in dosette boxes, as it was considered to go faster and save time. Despite the routine of sending everything expected to accompany the patient, RNHHCs often reminded RNHs to send everything based on the experience that something was easily missed.

Everything is supposed to be ready, the patient has already left the ward. And then there’s a call from the district nurse, things like that, that can also… of course that happens. Then it could be that there was a lot to do or it went too fast or that it has… well, anything can happen, that you forgot to send something, or… things like that. (P20H)

### Timing the departure

RNs in both organizations strived for an early departure time in the day from the hospital to enable reception at home. The distances from the hospital to the patient’s home entailed the need for this since the journey was time-consuming. The patient would preferably leave the hospital before lunchtime since it could take the whole day to travel home.

We try to keep in mind that they are residents in sparsely populated rural areas; well then, they may need to be discharged at eleven o’clock from us because it takes the rest of the day to get home. (P4H)

Dealing with sharp deadlines creating stressful discharges was thus something faced by the RNHs connected to patients living far away from the hospital. The most common mode of transport was carpooling in patient transport at one of three fixed times of the day. The patient transport took detours through the small villages along the way to drop off patients in various locations, which further extended the travel time. Poor maintenance of gravel roads and roadblocks placed demands on the patient transport driver’s local knowledge of the rural area. The transport was perceived as a risky element based on previous incidents involving orthopaedic patients being insufficiently relieved of pain during a bumpy journey, the patient lacking home keys, or dementia patients being taken to the wrong address.

We have some incredibly bad roads, where they sent home a patient with an upper arm fracture, this is several years ago, the patient was in deadly pain before he got home, because there were so many holes in the road that it was impossible, sitting in a taxi together with someone else […] something like that, I think, if you end up in an outer sparsely populated area, you have to think about who you send home and in what way. (P10HHC)

Seeing the whole of the CT, including the mode of transport, was therefore considered necessary to reduce the risks of patient suffering. The few times that a patient could be picked up by relatives were considered the best since it was perceived to be an assurance that the patient would reach home safe and secure.

What has happened is that the patient transport has left a patient who has been older and maybe has a little memory loss. Outside, all winter, she didn’t have a key and couldn’t get in. And then there has been a breakdown in communication, when such events occur. (P20H)

RNHHCs were trying to meet the patient at home early, preferably in connection with the return home. They were to make sure that medicines were correctly marked and sorted from the ward because what was not done correctly would otherwise spill over to the receiving RNHHC. They felt responsible for patient safety outside the hospital walls and had previously experienced incidents when they had not been there early enough. The early meeting enabled re-evaluation of the patients’ care needs, due to the wards’ difficulties in doing this. The early meeting at home could thus reduce the risk of delayed identification of mistakes, discomfort of the journey home or that the patient would have gone home with under-planned interventions. RNHHCs had at times received a report that the patient was in the same condition as prior to hospital admission, while actually they judged the patient as being in a worse-than-usual condition. They also had the experience of having received mixed messages or incorrect information, compelling them to form their own opinion of the patient. In the early meeting, it thus became clear whether the patient was discharged prematurely, or with insufficient planning. They could then try to reschedule efforts at home, arrange a place in short-term accommodation or send the patient back to the hospital. Some always wrote an incident report on such occasions, while others claimed they probably should do it more often. The early reception enabled an evaluation of how well the CT had gone.

I usually start by calling in and protesting and saying that now you made a mistake again, I’ll write an incident report. At the same time, I plan which assistant nurses I have in service who can go and catheterize. (P10HHC)

## Discussion

This study aimed to provide a deeper understanding of what RNs perceive as the main concerns in CTs from hospital care to home healthcare in rural areas, and how they handle these during the CT process. The substantive theory risk-reducing in complex CTs explains the importance of the core category *Actively communicating to reduce patient safety* risks, to handle the main concern *Care coordination in a complex process*. The active communication involved a constant interaction between the categories *Collaborating on expected care needs, Anticipating obstacles* and *Timing the departure*. Failure to perform those activities were associated with increased risk of poor patient outcomes in several respects. The findings show how communication and collaboration among healthcare staff are key factors for the quality of CTs (Carman et al., 2021; Coleman & Boult, 2003) and organizational pressures such as bed availability influence the process (cf.Carman et al., 2021; Åhsberg, 2019). Early planning for discharge is an effective way of approaching the length of stay at the hospital, thereby managing the shortage of hospital beds (Neale et al., 2020). This agrees with the substantive theory presented, explaining the value of RNs’ proactive stance as a way of reducing risks to handle the complex CT process, not least in rural areas where long distances were perceived to mean longer time from decision to execution. This study also adds that the pressure caused by the lack of beds is a reason for RNs taking this proactive stance.

Previous research (Petersen et al., 2019) shows that RNs in urban areas have patient safety as a goal in CTs from hospital to home. This is in accordance with the substantive theory presented, which also show that rurality may bring additional challenges in CTs, shown also by previous research (Henning-Smith et al., 2021). According to Fawcett (1984), the meta paradigm of nursing is built on the four central concepts of person, environment, health and nursing. Since the environment is one of these, the rural context bringing unique challenges may not be surprising. Patients’ safety and health as a goal may be considered a driving force for the core category, and the results of the RNs’ actions are thought to benefit the frail elderly and their relatives. This could be associated with the nursing process, not the least when adding communication as a sixth pillar (Burns et al., 2010).

The substantive theory explains the importance of RNs prioritizing to be a step-ahead in all their actions, since otherwise they risk complications such as prolonged hospital care or patient suffering. CTs can be a stressful and troublesome experience for frail older people (Andreasen et al., 2015; Lindblom et al., 2020), as well as for their relatives (Lindblom et al., 2020). In *Collaborating on expected care needs*, the assessments of future care needs by the patient, RNHs, assistant nurses on the ward and the welfare officer are explained, as well as the assessment of reasonableness by RNHHCs. The difficulty of these assessments is one reason why *Timing the departure* is such an important action, since RNHHCs prioritizing an early meeting at home and revising assessment of care needs presupposes RNHs strive for an early departure from the hospital, due to long distances. Correspondingly, *Anticipating obstacles* interacts with the other actions since sufficient knowledge and staffing in home health care is based on the assessments made in *Collaborating on expected care needs*, while evaluation of *Anticipating obstacles* also takes place in *Timing the departure*. Thus, according to the substantive theory, there is a constant interplay between these critical actions of the transition process.

Further, the results explains that RNs reduce patient safety risks by actively communicating, doing so by activities such as collaborating, anticipating, and timing. These activities connect interactively to each other through the theoretical code *constantly prioritizing*. The actions were related to both expected and apprehended scenarios concerning the patient being transitioned, as well as to the other and next patients in the hospital ward. This can be interpreted as their acting on the ethical code for nurses in the matter, taking responsibility for those in need of care in the present and the future (International Council of Nurses, 2021). It may be even more understandable relative to the process theory. As per the result, RNs act and predict based on previous experiences, which is closely related to what Hernes (2014) described as organizing by contingency plans. According to Hernes (2014), processes are multifaceted and shaped by the choices made concerning experiences related to the past, present and potential future, which, as in the results of this study, makes processes complex.

Risks overlooked in one stage can be solved in the next step—or cause patient suffering, while the process is more cyclical than horizontal. This can be explained by the theoretical code “Constantly prioritizing”. The actions may conceivably be founded on nurses’ fundamental responsibility to promote health, prevent illness, restore health, and alleviate suffering (International Council of Nurses, 2021). Individual collaboration is related to current organizational policies, which, according to Littzen et al. (2020), form parts of the RNs daily practice. RNs depend on the ability to delegate tasks to assistant nurses to enable CTs, which highlights the importance of sufficient nursing staffing to pursue patient safety. This is as per the results and accordingly with Mccullough et al. (2020) especially in rural areas where the RNs must travel a long way to reach the patients and other healthcare providers. Sufficient staffing is a prerequisite for RNs’ teamwork both within the profession and with other professions. However, in line with previous research (Henning-Smith et al., 2021; Norlyk et al., 2020; Taylor et al., 2020), staffing challenges such as difficulty in recruiting staff and keeping them updated with condition-specific care are tangible challenges in a rural context.

These results are similar to previous research on discharges in rural areas, showing that communication, infrastructure, and timing are prominent factors affecting CTs (Henning-Smith et al., 2018). For RNs to reduce risks in CTs, educational institutions must recognize active communication between healthcare organizations as a core competency for healthcare professionals such as RNs. Also, policymakers must understand the critical role of quality in CTs and how CTs affect the outcomes of the frail elderly with complex care needs (Coleman & Boult, 2003). According to the WHO (2016), the location of the patient’s home is a factor that may affect CTs. This means policy makers must bear in mind what additional risks the rural environment may bring and in what ways rurality may affect the organization of CTs, such as presented in this study, to optimize the quality thereof to meet the needs of the frail elderly and their relatives.

This study is a novel contribution to the knowledge about CTs in rural areas. The authors do not claim to have explained all the temporal sequences of the complex CT process, but the reported sequences build the core category. More temporal sequences probably exist than explained in this study; accordingly, future research should investigate which more prominent sequences can conceivably affect the quality of care during CTs in rural areas, by further investigating these and other actions. Since these CTs are so complex, more research is needed to fully understand and improve the guidelines for inter-organizational collaborations, team collaborative processes and environmental structures.

## Strengths and limitations

Several methodological aspects of this study are interesting to discuss in relation to the Constructivist Grounded Theory method and Charmaz’s (2014) four criteria for quality: credibility, originality, resonance, and usefulness. The design itself may be considered a strength of this study, since the Constructivist Grounded Theory is useful when studying processes (Charmaz & Thornberg, 2021). How well the method matches the research question has a major impact on the quality of Grounded Theory studies (Chun Tie et al., 2019). Including participants from county hospital care and municipal home healthcare can also be considered a strength, since comparing actions between two different organizations can help clarify organizational processes and structures (Charmaz, 2014).

Since there is no single optimal definition of “rural” (Hawley et al., 2016), the authors have, to the best of their ability, given a thick description of the context in which the study was conducted. Such a description of the context is a prerequisite for thick interpretation, which in turn is required for the study’s credibility and resonance (Ponterotto, 2006). It may optimize the reader’s understanding of the studied phenomenon and enhance the usefulness of the study findings (Charmaz & Thornberg, 2021; Charmaz, 2014). The usefulness of a study depends on the extent to which the findings contribute to knowledge and inspire new ideas for research in other areas (Charmaz & Thornberg, 2021); to ensure this, accuracy and clarity in the analysis and presentation of the study have been sought.

Systematic comparisons were made between the data and the categories and data collection was guided by the endeavour for theoretical saturation. The probing questions were very important in the construction of the main concern and the core category since they became more theoretical, as data collection and analysis progressed. The memos which helped the analysis and directed following theoretical sampling are, according to Chun Tie et al. (2019), highly important to the quality of a Grounded Theory study. The first author’s sharing of memos with the other authors may be considered a methodological strength since Charmaz’s (2014) recommends this to maintain reflexivity.

The reason for the uneven distribution of participants from the hospital and home healthcare is unknown and, for ethical reasons, has not been questioned or investigated further. The possible reasons may be several, but since one hospital clinic manager declined due to a high workload while RNs testified to a stressed and pressured situation at the hospital, contributing to the main concern, it could be one of the possible reasons. Since the study aimed at an increased understanding of the process and theoretical saturation was achieved, the credibility and resonance still seem satisfactory (Charmaz, 2014).

The participants being offered the choice of the time and mode of the interviews meant that facial expressions, body language and gesticulations could not be observed in telephonic interviews. However, no differences in content richness between the interview forms were identified. It may rather be considered a strength since according to Elwood and Martin (2000), it may optimize the participants’ comfort to speak openly and feel empowered in the interaction with the researcher.

The RNs’ recalled memories, experiences and perceptions could be captured during the interviews. Additional data collection methods, such as studying documentation or performing observations, could have given an even better understanding of their actual behaviours during the transition process. However, the data collection can be regarded as solid since theoretical saturation was achieved. In the Grounded Theory, this strengthens the analysis and credibility (Charmaz, 2014), and thereby, the quality of the study (Charmaz & Thornberg, 2021). Additionally, the codes and categorizations were discussed among the authors, enhancing the trustworthiness of the study (Charmaz, 2014).

The findings in this study have been reviewed internally by specialists (outside the research team with subject knowledge and experience at a script seminar), who recognized what emerged while also finding the results to offer new insights. Their reactions could speak for the study’s resonance and originality. According to Charmaz’s (2014), it means that the results should not only be comprehensible to people operating in a context similar to the participants like these specialists but also bring new insights. The originality can also be considered enhanced by the fact that there seems to be sparse research in this context. Therefore, these findings are an important contribution both theoretically for the current research field and socially, if used as a basis for improvement work (Charmaz, 2014).

## Conclusion

This study is a contribution towards an increased understanding of how RNs in rural areas can proceed when they care for a patient in a risky CT process from hospital to home healthcare. It emphasizes the importance of clarity within and between organizations in what the actors must relate to. This study explains the challenges posed by unclear agreements, lack of care beds and previous experience of mistakes. In addition, it shows specific, rural context-related challenges such as long distances, long delivery times and experiences of increasingly tight deadlines. These aspects must, as per the result, be considered throughout the whole CT process, at the hospital and home healthcare where the RNs work, as well as by the organizations’ healthcare leaders who develop collaboration agreements, and guidelines on how these CTs are to be performed. The context in which the patient lives and where care is being delivered must thus be considered at every step, for the delivery of good quality care. This study also highlights the importance of, not only as individuals but as a team, within and between organizations, trying to handle problems that arise through this complex CT process. Hence, it can be considered important in rural areas to optimize factors that can be facilitated through context-adapted organizing, such as clear guidelines to adhere to, adequate communication tools and sufficient staffing, as a basis for collaboration.

## Data Availability

Access to the data set can be obtained by e-mail to the corresponding author upon reasonable request.
